# Automated Computer-Assisted Image Analysis for the Fast Quantification of Kidney Fibrosis

**DOI:** 10.3390/biology11081227

**Published:** 2022-08-17

**Authors:** Esteban Andrés Sánchez-Jaramillo, Luz Elena Gasca-Lozano, José María Vera-Cruz, Luis Daniel Hernández-Ortega, Adriana María Salazar-Montes

**Affiliations:** 1 Instituto de Investigación en Enfermedades Crónico-Degenerativas, Centro Universitario de Ciencias de la Salud, Universidad de Guadalajara, Guadalajara 44340, Jalisco, Mexico; 2 Instituto de Nutrigenética y Nutrigenómica Traslacional, Centro Universitario de Ciencias de la Salud, Universidad de Guadalajara, Guadalajara 44340, Jalisco, Mexico; 3 Centro de Investigación Multidisciplinario en Salud, Centro Universitario de Tonalá, Universidad de Guadalajara, Tonala 45425, Jalisco, Mexico

**Keywords:** kidney, fibrosis, morphometrics, image analysis, computer-assisted, Masson’s Trichrome, CellProfiler, ImageJ

## Abstract

**Simple Summary:**

Chronic kidney disease is a health problem in which the kidneys cannot function normally. Thus, they cannot filter blood effectively and cause waste accumulation in the organism, leading to serious health problems. Researchers use animals as models to replicate the human body’s behavior to understand this disease. In these studies, it is essential to evaluate the percentage of fibrosis (growth of fibrotic tissue similar to a scar in response to damage) to know the degree of kidney damage. Some researchers use programs to make the evaluation of fibrosis easier. However, this analysis is time-consuming because it needs to be made one image at a time and there are hundreds of samples in an animal model study. Here, we explain a method to conduct the same analysis but in a faster automated way with the assistance of a computer and a software package called CellProfiler™. The percentage of fibrosis using CellProfiler™ is similar to that obtained with the most widely used software for this kind of analysis called ImageJ. With the help of this approach, researchers can make more studies faster and easier and find new antifibrogenic therapies to address the common and worldwide health problem caused by chronic kidney disease.

**Abstract:**

Chronic kidney disease (CKD) is a common and worldwide health problem and one of the most important causes of morbidity and mortality. Most primary research on this disease requires evaluating the fibrosis index in animal model kidneys, specifically using Masson’s trichrome stain. Different programs are used to calculate the percentage of fibrosis; however, the analysis is time-consuming since one image must be performed at a time. CellProfiler™ is a program designed to analyze data obtained from biological samples and can process multiple images through pipelines, and the results can be exported to databases. This article explains how CellProfiler™ can be used to automatically analyze kidney histology photomicrographs from samples stained with Masson’s trichrome stain to assess the percentage of fibrosis in an experimental animal model of CKD. A pipeline was created to analyze Masson’s trichrome-stained slides in a model of CDK induced by adenine at doses of 50 mg/kg and 100 mg/kg, in addition to samples with the vehicle (75% glycerin). The results were compared with those obtained by ImageJ, and no significant differences were found between both programs. The CellProfiler™ pipeline made here is a reliable, fast, and easy alternative for kidney fibrosis analysis and quantification in experimental animal models.

## 1. Introduction

Chronic kidney disease (CKD) is a common and worldwide health problem and one of the most important causes of morbidity and mortality due to diverse concurring diseases, including type 2 diabetes mellitus, hypertension, nephritis, and lupus, among others [1]. Kidney fibrosis is CKD’s main and end-stage manifestation; it refers to both tubulointerstitial fibrosis and glomerulosclerosis [2]. The histopathology of kidney fibrosis includes the exacerbated deposition of the extracellular matrix (ECM), immune cell infiltration, glomerular regression, tubular expansion with epithelial cell loss, accumulation, activation, and generation of myofibroblasts via partial epithelial-to-mesenchymal transition [2,3,4]. Tubular epithelial cells are the main kidney structure damaged in tubulointerstitial fibrosis in CKD [3,4].

In order to know how fibrosis leads to damage to the kidney structure, various experimental animal models are used because they emulate the damage, molecules, and biological processes that occur in the human body, especially in the kidney. All these models use a significant number of histological samples for morphometric analysis. Quantifying fibrosis is essential for knowing the degree of injury in the kidney and measuring the percentage of ECM in a trichrome-stained sample. Since kidney fibrosis is an area of extensive research for evaluating new antifibrogenic therapies, scientists need efficient and easy-to-use methods for quantifying fibrosis in those experimental models. Many pathologists use a semiquantitative method, such as a score, according to the affected area and pattern of the injury in order to classify them into stages. The limitation of this method is its subjectivity since it depends on the visual interpretation of an expert pathologist, and it is not sensitive enough when there are small changes between samples.

Quantitative methods include taking photomicrographs of trichrome-stained samples and analyzing them with specialized image software. This approach is much easier, faster, and reproducible, is sensitive enough, and does not require a pathologist to be able to use it. Some programs such as Image-Pro, QuPath, and ImageJ can be time-consuming due to the analysis being made one image at a time or via the creation of macros that are not customizable or changed once created depending on the set of images analyzed. To overcome this problem, automated software can analyze hundreds of images in a fraction of the time and effort compared to similar programs for image analysis. One of these is CellProfiler™, a free, open-source, and modular program.

CellProfiler™ is a program designed for and by biologists to measure cells and analyze data obtained from biological samples [5,6]. Some of its best features are modularity, ease of use, and the ability to automatically process hundreds or even millions of images using pipelines that can be exported to databases and used by everyone [7]. The software (Stable version 3.1.9) can be installed on Windows^®^ and Mac^®^ computers and is available at www.cellprofiler.org (accessed on 7 July 2022). Pipelines are small programs created in the graphical user interface (GUI) of CellProfiler™ that are easy to create, save, and share [6]. They sequentially use modules for specialized image analysis with processing such as object form identification, color balance and extraction, object measurement, area quantification, and data exporting. This study aimed to provide a tool for the simultaneous and automatic analysis of multiple photomicrographs of kidneys from different murine models of CKD stained with Masson’s trichrome stain for the rapid quantification of fibrosis. The pipeline made and described here can be downloaded from the official program website (in About/Published Pipelines) or from the Supplementary Material and be used freely.

## 2. Materials and Methods

### 2.1. Animal Model of Kidney Fibrosis

The experiments with animals were conducted in compliance with the guidelines of animal use and handling of the Universidad de Guadalajara. Male C57BL/6 mice with an average weight of 20–25 g were housed in cages with food and water *ad libitum* in a controlled ambient temperature and humidity with a 12-h light cycle.

Adenine, purchased from Sigma Aldrich (St. Louis, MO, USA), was orally administered daily using 75% glycerin as a vehicle at a dose of 50 mg/kg and 100 mg/kg for 28 days. The Control group was administered the vehicle only. This model induces tubulointerstitial fibrosis through the accumulation of adenine crystals in the kidney, generating inflammation and the expression of profibrogenic genes [8,9].

### 2.2. Histopathology Analysis

Samples of both kidneys were taken after 28 days of fibrosis induction, fixed in 10% paraformaldehyde, and embedded in paraffin. Kidney tissue samples were sectioned at 5 μm thickness and stained with Masson´s Trichrome for ECM deposition visualization. Four samples from the right and left kidneys were taken from each animal of every group.

### 2.3. Image Digitalization and Analysis

Twenty non-overlapping random fields photomicrographs per sample were taken using an optical microscope with 200X magnification connected to a PC with the Future WinJoe v.1.6 image acquisition software (Future Optics Sci. & Tech. Co., Hangzhou, China). The format obtained with the image acquisition software was JPG and this format was used for all the loaded images in the pipeline. CellProfiler™ accepts a wide range of image formats such as JPG/JPEG, BMP, IMG, PNG, and TIF/TIFF, among many others. It is recommended to use some lossless image format as TIF/TIFF when acquiring the images, but the results do not change if some format or other is used when analyzing them with this pipeline, at least when all images have the same format. The resolution used for capturing the images with the image acquisition software was 96 ppi, with dimensions of 1024 × 768 pixels (1.3 megapixels resolution).

### 2.4. Pipeline Creation

CellProfiler™ v.3.1.9 (Broad Institute, Cambridge, MA, USA) was used to create the pipeline described below. The program uses pipelines that contain sequential steps for image analysis. Every step consists of a module with different options and values adapted to fit an experiment. The entire process of making the pipeline for analyzing kidney fibrosis samples is described step by step below.

### 2.5. Statistical Analysis

Data analysis was performed using the GraphPad v.5.0 software for Windows (GraphPad Software Inc., San Diego, CA, USA). The Student’s *t*-test was used for data comparison between two unpaired groups. Data are presented as the mean ± SD. A *p*-value < 0.05 was considered statistically significant.

## 3. Results

**Figure 1:**
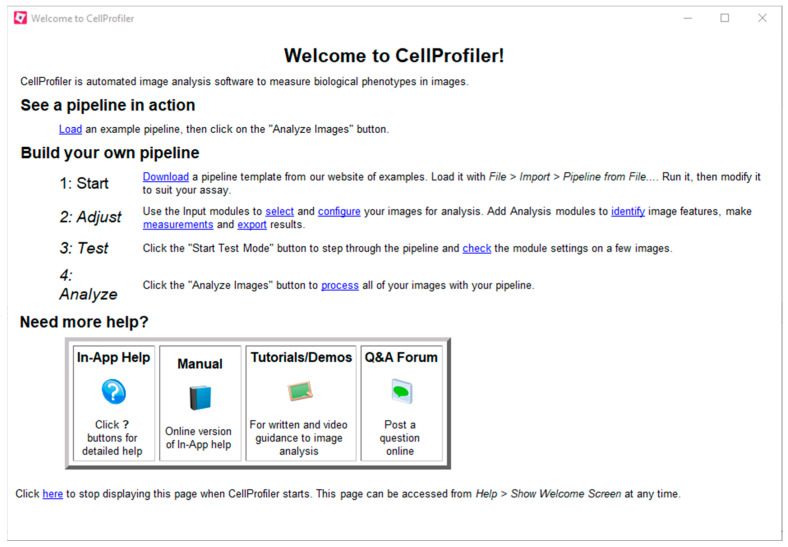
Welcome Screen of CellProfiler™ v.3.1.9 shows the options to see examples of pipelines or create a new one with help and tutorials.

When the program opens, the Welcome Screen appears; here, a new pipeline can be created (Figure 1). To load the pipeline, select “Open” and then the name of the file (KidneyFibrosisComplete v2.0.cpproj, File S1). At the bottom of the program window, there are buttons to add (+), remove (−), or rearrange (^^^, ˇ) a module of the pipeline. Additionally, the mode “Test” allows for the user to start the test mode by stepping through the pipeline modules one at a time by user input to check if the module settings are right, testing one or few images. The “Analyze images” mode processes all the loaded images automatically when everything is right.

**Figure 2:**
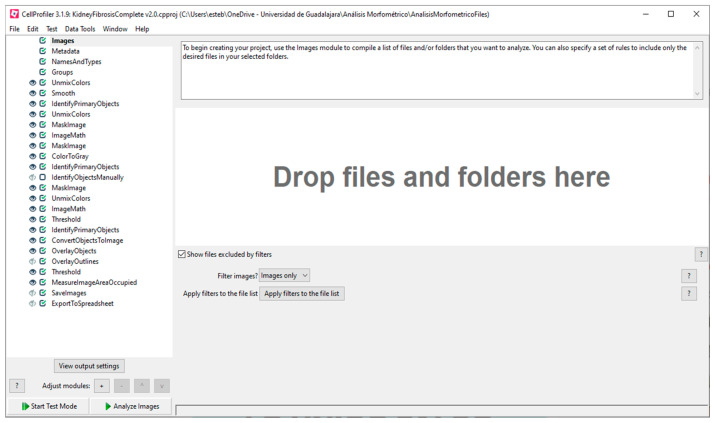
CellProfiler™ pipeline for the analysis of kidney fibrosis.

The loaded pipeline for the analysis of kidney fibrosis shows all the required modules, including the first four modules by default: *Images*, *Metadata*, *NamesAndTypes*, and *Groups* (Figure 2). With the *Images* module, a list of images can be compiled to be analyzed, including options to apply filters. The images are loaded via dragging and dropping them in the area labeled “Drop files and folders here”. If the loaded images have metadata, that information can be extracted and included in the experiment with the *Metadata* module. The *NamesAndTypes* module allows assigning a name to the loaded images to be used by the program such as the image type (color, grayscale, or binary mask). The *Groups* module is optionally used to separate images into different groups and subsets that will be processed independently of the others based on their metadata (different image format, source, type, or screening batch date).

**Figure 3:**
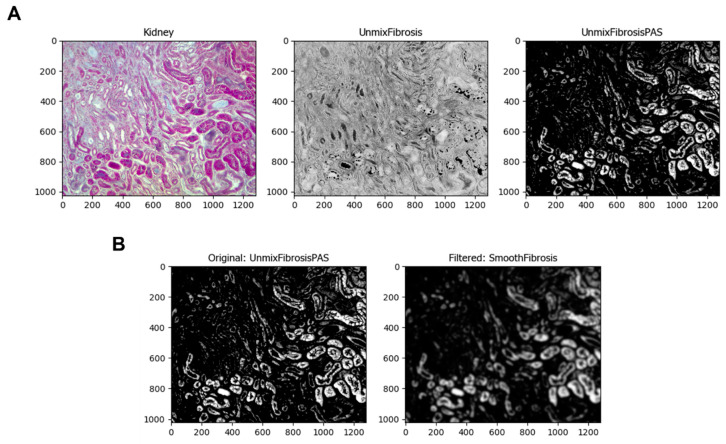

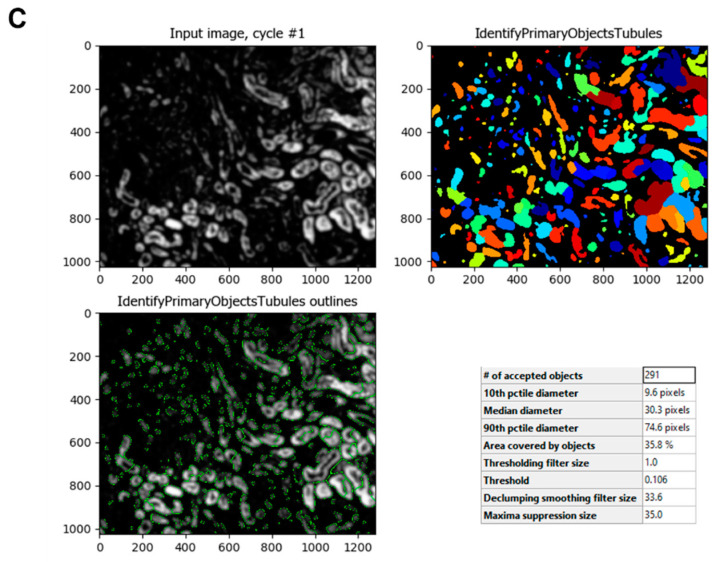
Identification of tubules. (**A**) The *UnmixColors* module separates the image of one or more stains (here, Hematoxylin and PAS as primary and PAS as secondary). (**B**) The *UnmixColors* module separates the image of one or more stains (here, Hematoxylin and PAS as primary and PAS as secondary). (**C**) The *IdentifyPrimaryObjects* module is used to identify cell structures, in this case, it is used for the identification of kidney tubules.

The next module is *UnmixColors*, where the program can separate the colors of a specific stain using its absorbance values. This pipeline uses Hematoxylin and PAS (periodic acid Schiff) as primary and PAS as secondary (Figure 3A). The *Smooth* module smooths the images with a particular filter, in this case, the Gaussian Filter. The image processing of this module blurs the objects, delineating the lighter ones to be identified. These objects are the normal renal tubules stained by Hematoxylin, one of Masson’s dyes. The image that this module took was *UnmixFibrosisPAS*, the resulting image of the *UnmixColors* module (Figure 3B). To identify the normal tubules in the image, the *IdentifyPrimaryObjects* module is used. Within this module, there are several settings where the typical diameter, threshold method and scale, and the method the module uses to distinguish objects can be entered. Depending on the image, these settings must be changed. The resulting process image shows the identification of the objects in green circles and the area of those in different colors. A table with various values is also displayed in the pop-up window (Figure 3C).

**Figure 4:**
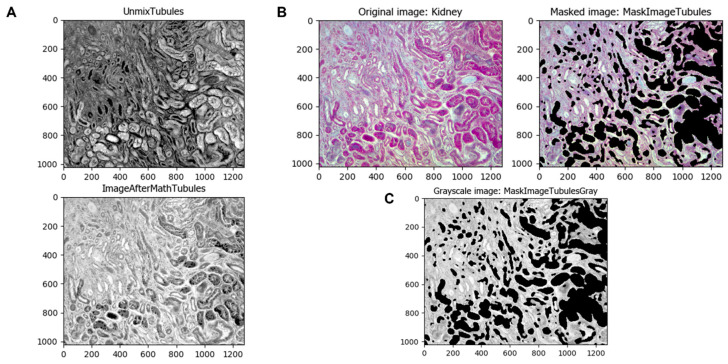
Separation of identified tubules from the original image. (**A**) The *ImageMath* module can be used to make operations such as inverting the image. (**B**) The *MaskImage* module masks the original image with the tubule-less image. (**C**) The *ColorToGray* module changes the color image to a grayscale one.

To intensify the normal kidney tubules and later separate them from the rest of the image, it is first inverted using the *ImageMath* module (Figure 4A). This module can apply other math operations to images such as add, subtract, multiply, divide, etc. The image obtained is masked with the original one using the *MaskImage* module to obtain a tubule-less image and convert it to grayscale with the *ColorToGray* module (Figure 4B,C).

**Figure 5:**
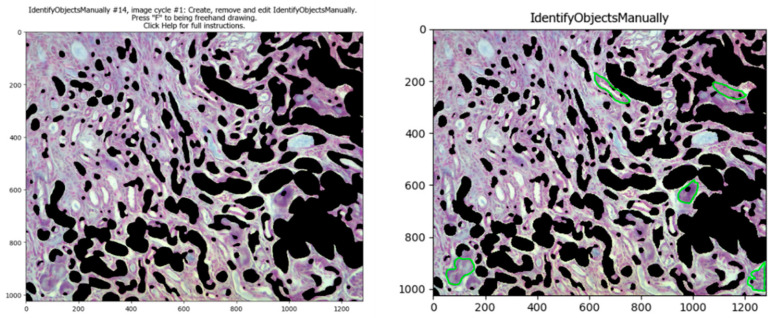
Manual identification of objects. The *IdentifyObjectsManually* module, if enabled, lets the user select objects or areas in the image (manually selected areas are in green after the freehand drawing).

Some objects such as high stained areas or some glomeruli are difficult to select automatically and do not need to be taken into consideration for an analysis of tubulointerstitial fibrosis. If this is the case, the pipeline has the *IdentifyObjectsManually* module; when it is enabled (clicking the check mark), the program lets the user select them by freehand drawing those areas. This module requires manual input, but only when it is strictly necessary; in those cases when this module is necessary to enable, is recommended to only analyze that image, and later analyze the others automatically with this module disabled (clicking the check mark again). Later, that image can be masked with the previous image with the *MaskImage* module (Figure 5).

**Figure 6:**
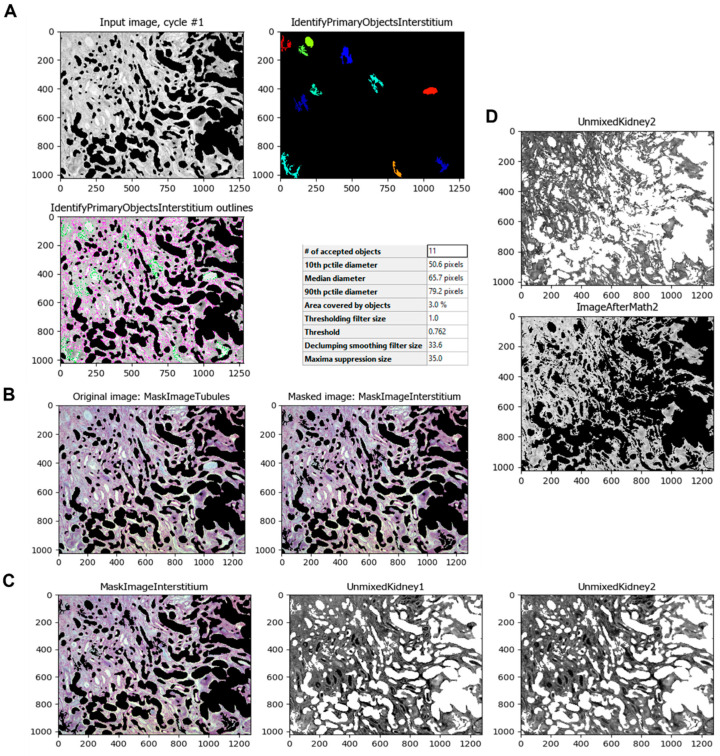
Identification of small areas of the renal interstitium and inside tubules, and separation of Masson’s trichrome stain colors. (**A**) The *IdentifyPrimaryObjects* module identifies the normal renal interstitium and other non-fibrotic areas. (**B**) The *MaskImage* module masks the tubule-less image with the normal renal interstitium image. (**C**) The *UnmixColors* module separates the image of the stains Methyl Blue (collagen) and Ponceau-fuchsin (Masson’s Trichrome red counterstain). (**D**) The *ImageMath* module inverts the image obtained by the unmixing process.

The same process is applied to the renal interstitium since the experimental model in this study produces tubulointerstitial fibrosis. Using the *IdentifyPrimaryObjects* module, the program can select those areas of the normal renal interstitium and additional non-fibrotic areas as the intratubular space for better results (Figure 6A). Once the program identifies those objects, the module *MaskImage* (Figure 6B) is applied as in the identification of the renal tubules. The module *UnmixColors* is then used to separate the stains used in the samples, such as Methyl Blue (for collagen) and Ponceau-fuchsin (Masson’s Trichrome red counterstain) (Figure 6C). Using the *ImageMath* module, the resulting image is inverted (Figure 6D).

**Figure 7:**
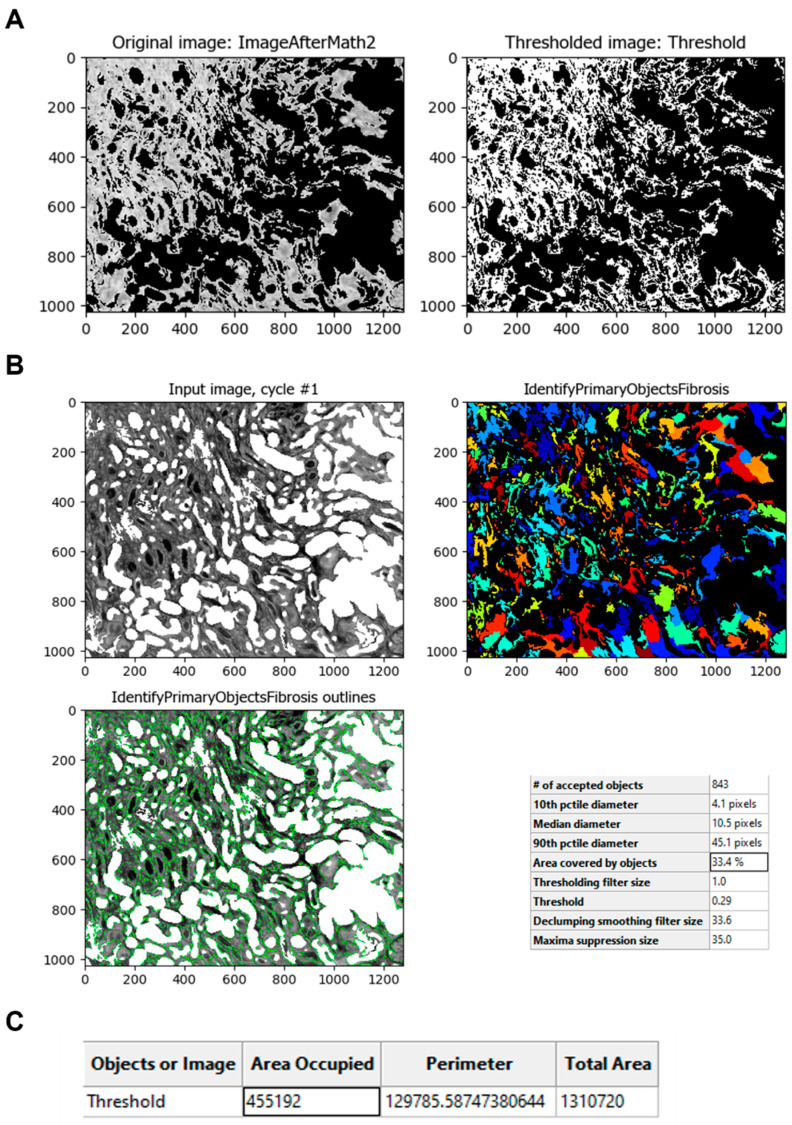
Fibrotic area identification and calculation. (**A**) The *Threshold* module calculates the threshold value to obtain the fibrotic area (white). (**B**) The *IdentifyPrimaryObjects* module identifies the fibrotic areas and gives a pre-value of the fibrotic percentage (boxed). (**C**) The *MeasureImageAreaOccupied* calculates the area of the fibrotic objects in the image (boxed).

The *Threshold* module is then used to obtain an image that can be processed to identify the fibrotic areas (Figure 7A). This module calculates the threshold value of the unmasked pixels of the image. The value obtained is used to classify pixels above and below; the pixels above are treated as foreground, and the pixels below are treated as background. This value can be calculated automatically or can be adjusted by a correction factor to apply another method or strategy option to adapt more precisely across all images. The resulting process creates a binary image that shows the fibrotic area in white (1) and the background in black (0). With the resulting image, the fibrotic areas can be identified using the *IdentifyPrimaryObjects* module (Figure 7B). Once the image is thresholded, the program can measure the fibrotic area using the *MeasureImageAreaOccupied* module (Figure 7C).

**Figure 8:**
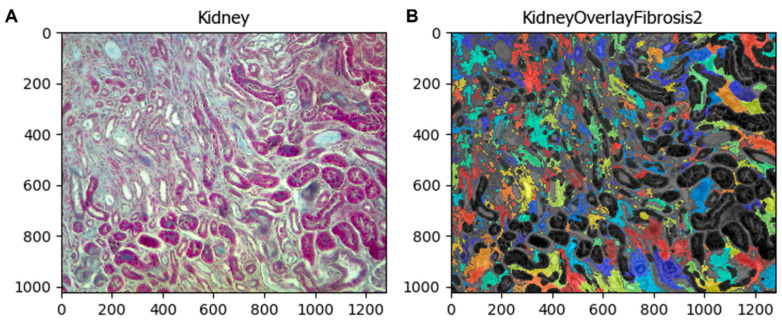
Comparison between the fibrotic area with the original image. (**A**) Original image, (**B**) merged image.

If the user wants to compare the fibrotic area identified by the pipeline with the original image, the module *OverlayObjects* merges both images, maintaining the objects colored (fibrotic area) for easy comparison between them (Figure 8).

The fibrotic area image and the data obtained can be exported as images and spreadsheets with the *SaveImages* and *ExportToSpreadsheet* modules, respectively. Once the exporting is ready, the percentage of fibrosis can be calculated using the formula: %fibrosis = (area occupied fibrosis/total area) * 100. The spreadsheet obtained has many columns filled with data; the only columns needed for calculation are “AreaOccupied_AreaOccupied_ThresholdFibrosis” (first column) and “AreaOccupied_TotalArea_ThresholdFibrosis” (third column). The percentage of fibrosis in the processed image was 34.73%.

### Comparison with ImageJ

**Figure 9:**
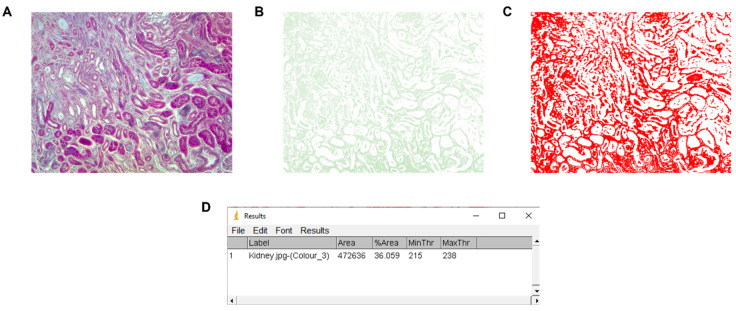
Fibrotic area calculation using ImageJ. (**A**) Original image, (**B**) green channel, (**C**) thresholded, and (**D**) measured fibrosis area and percentage with the minimum and maximum threshold values.

To compare the results obtained with our pipeline, we also performed the analysis with the most widely used image processing software, ImageJ v.1.53 (National Institutes of Health, Bethesda, MA, USA), which employed the “Colour deconvolution” plugin [10]. With the ImageJ program, the fibrosis index measured (fibrotic area) was 36.06% (Figure 9).

**Figure 10:**
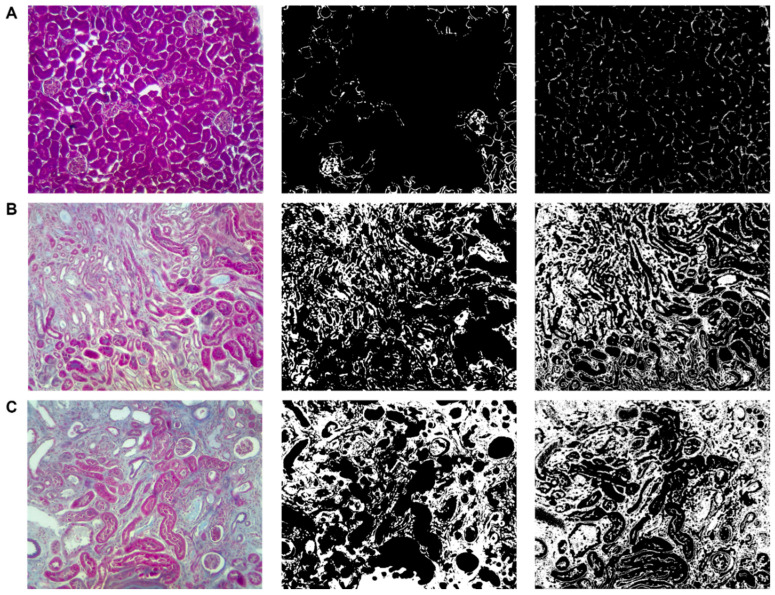
Fibrotic area measurement of kidney samples. From left to right: original image, CellProfiler™ pipeline result, ImageJ result. (**A**) Treated with vehicle, (**B**) treated with adenine (50 mg/kg), and (**C**) treated with adenine (100 mg/kg). Magnification of 200×.

The image used to calculate the fibrosis percentage was obtained from a sample of a kidney treated with 100 mg/kg of adenine. The fibrosis area was also evaluated using samples treated with 50 mg/kg of adenine and the vehicle as control (Figure 10).

**Figure 11:**
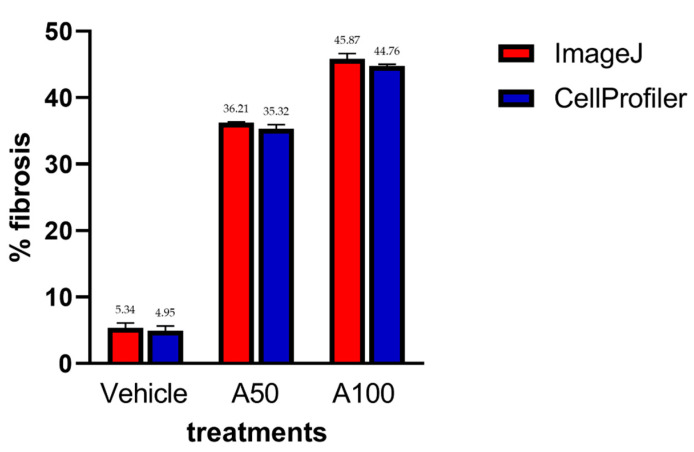
Fibrotic area measurement between ImageJ and CellProfiler™ with different treatments. No significant statistical differences were found between the programs. Mean ± SD (*n* = 5). Vehicle: 75% glycerin; A50: adenine (50 mg/kg); and A100: adenine (100 mg/kg).

No significant statistical differences were found between the fibrosis percentage in the measured samples using both CellProfiler™ and ImageJ programs (Figure 11).

## 4. Discussion

In recent years, CKD has risen as one of the most prominent health problems in the world [1]. The last stage of kidney damage by CKD is fibrosis, an exacerbated extracellular matrix deposition that decreases kidney function [2]. In this sense, an increased number of in vivo studies are being performed in the field of nephrology; most of them report the assessment of the percentage of fibrosis in the kidney tissue using hundreds of images on experimental animal models [4,8,9,11,12,13,14] or renal biopsies [15]. However, the measurement of fibrosis in that scenario is laborious when it is done using a manual approach, such as a score determined by a pathologist or employing a non-automated computer program [4,11,16].

Recently, a computerized approach to image analysis has been gaining prominence, new methods are being developed, and some are more sophisticated than others. Although there are better approaches such as artificial intelligence [17] and machine learning [18] that use convolutional neural networks trained to learn how to interpret the results of a given sample, reproducing the pathologist visual assessment [19,20,21,22], there are also more straightforward approaches such as image processing software. One of the most used is the free software ImageJ [13,23,24,25,26,27] and, more effectively, automated software such as CellProfiler™.

The pipeline presented in this article helps analyze kidney fibrosis in Masson’s Trichrome stained images and quickly obtain the fibrotic area without needing complicated scripting or knowledge of specialized software. Our approach gives similar results to the ones obtained with ImageJ, but automatically, with the option of batch processing hundreds of images. The percentage of the fibrotic area is more accurate than the one obtained with ImageJ because the pipeline can identify renal structures as tubules, glomeruli, or vessels with high stained areas. Therefore, it is normal to expect a higher fibrosis percentage using ImageJ than the pipeline; otherwise, ImageJ requires more time fine-tuning every image to eliminate high stained areas with a background correction step for subtracting them. Another benefit of this pipeline is the plethora of options in every module to adapt more precisely to different staining qualities and intensities, some of which ImageJ does not have. Even though ImageJ has the macro ability, it applies the same values, especially in the threshold calculation, to all samples since the macros are pre-built procedures that run as defined steps that do not adapt to every image.

On the contrary, CellProfiler™ modules adapt automatically to variations in images. Furthermore, the threshold values are calculated depending on the intensity or other characteristics of the image. All of this helps to improve scientific reproducibility [6].

Regarding time, using ImageJ, the analysis can take around 5–10 min per image sample; the most time-consuming step is determining the threshold value. On the other hand, CellProfiler™ can analyze around 20–30 image samples in the same time, depending on the computer’s hardware specifications. Although the resolution does not greatly affect the results of the pipeline, the time required to analyze a high-resolution image increases dramatically. In this regard, resolution ranging from 1024 × 768 (1.3 megapixels) to 2048 × 1536 (3.1 megapixels) is ideal to analyze images with this pipeline to obtain fast results. In terms of the validation of the pipeline described here, it was applied to another work by our group [28] where we used 800 images of stained slides to evaluate the percentage of tubulointerstitial fibrosis on an animal model of CKD.

Even though this pipeline measures the fibrotic tissue area in stained slides only in those areas of tubulointerstitial fibrosis, it does not distinguish normal fibrotic tissue (connective tissue of basement membranes) from pathological fibrotic tissue. In this sense, workarounds can be made to overcome this limitation and challenge, such as subtracting the fibrotic area calculated previously from a control sample or combining Masson’s Trichrome with PAS staining. This method is made by deriving a differential staining area by subtraction morphometry [15,29] and using this pipeline on that derived image. Alternatively, polarized Sirius Red staining (specific for collagen types I and III) can be used.

It is important to note that the settings values of every module presented here must be changed to adapt to different image lots, depending on the intensity of the colors of the Masson’s Trichrome staining. Grouping images from the same animal or collection date is unnecessary because the pipeline modules adapt to different images obtained from different animals. On the contrary, other criteria such as animal condition, reagent lot, and staining process date can be important for grouping when the user needs to change the settings in some modules to better adapt to differences in staining. However, once one representative image is analyzed and the area of fibrosis correctly determined, the pipeline does all the processing work for the rest of the images in the lot. In this sense, it is essential to analyze the same group of images with the same image acquisition software options of the microscope or camera to avoid variability in staining intensity. The most important setting to change in the pipeline is the threshold correction factor in the *IdentifyPrimaryObjects* and *Threshold* modules.

This pipeline was created with the purpose of morphometrically analyzing samples taken from experimental animal models, especially murine models of CKD. It is limited to quantifying tubulointerstitial fibrosis and not glomerulonephritis or glomerulosclerosis in animal models because glomeruli are excluded for analysis. However, in the future, it is intended to obtain patient samples to validate and subsequently use them in human-derived samples. Additionally, we intend to expand its use and apply machine learning methods inside the pipeline to obtain better results quantifying kidney fibrosis.

## 5. Conclusions

The described pipeline was successfully used in an animal model of adenine intoxication to measure kidney fibrosis. This automated computer-assisted image analysis approach gives researchers a practical tool that is easy to use and reliable. It can be employed to obtain more precise and reproducible results in a fraction of the time and effort needed with other widely used image analysis software packages that require constant user intervention.

## Data Availability

The data presented in this study are available on request from the corresponding author. The pipeline made and described in this article can be obtained from https://cellprofiler.org/published-pipelines (accessed on 7 July 2022).

## Supplementary Materials

The following supporting information can be downloaded at: https://www.mdpi.com/article/10.3390/biology11081227/s1. File S1: KidneyFibrosis_Sanchez_2022.
