# Supplementary material for: Automated Computer-Assisted Image Analysis for the Fast Quantification of Kidney Fibrosis
Source: Biology (Basel). 2022 Aug 17;11(8):1227. doi: 10.3390/biology11081227 (PMC9404825; doi:10.3390/biology11081227)
Supplement: Supplementary file 1 [file biology-11-01227-s001.zip › biology-1844525-supplementary/biology-1844525-supplementary.pdf]

KidneyFibrosisComplete v2.0 Notes:

Image analysis for quantification of fibrosis from Masson's Trichrome stained slides using CellProfiler (https://www.cellprofiler.org). A quick description of every module used is listed below:

Images: Loads images to be analyzed. A folder with hundreds of images can be added.

Metadata: Metadata describing every image can be extracted.

NamesAndTypes: This pipeline uses the name "Kidney" to be recognized as the image name (do not change it).

Groups: Slits images into groups to be analyzed independently.

UnmixColors: This module can separate the colors of specific stains using their absorbance values.

Smooth: Smooths the images with a particular filter.

IdentifyPrimaryObjects: Identifies the tubules in the image. Values range from 50 to 160.

UnmixColors: Separates custom stains. In this module the pipeline uses "Hematoxylin and PAS" as primary stain and "Hematoxylin" as secondary stain. An estimate absorbance from an image can be loaded using "Estimate".

ImageMath: Inverts the original image to intensify the normal tubules.

MaskImage: Masks the original image with the unmixed images (without tubules). An inverted mask option is used.

ColorToGray: Changes the masked color image to a grayscale one.

IdentifyObjectsManually: Identifies manually objects that are hard to identify. This is an optional module that is disabled by default, if enabled, another window will appear to manually select objects.

MaskImage: Masks the grayscale image without tubules with the manually selected image.

IdentifyPrimaryObjects: Identifies other elements on the images such as renal interstitium, vessels, glomeruli, and high stained areas. Values range from 50 to 220. If the "*IdentifyObjectsManually"* module is enabled, please change the input image name from "MaskImageTubulesGray" to "MaskManually".

MaskImage: Masks the grayscale image without tubules with the last image obtained.

UnmixColors: Unmixes the Masson's Trichrome stains. In this module the stains used are "Methyl Blue" and "Ponceau-fuchsin". Remove the stains and change to "Custom" if you want to use another stain based on the absorbance values. An estimate absorbance from an image can be loaded using "Estimate".

ImageMath: Inverts the last unmixed image.

Threshold: Thresholds the inverted image using "Global" strategy and "Otsu" method with a correction factor of "0.9". Change these values accordingly to the images analyzed.

ConvertObjectsToImage: Converts the identified objects (fibrosis) to an image.

OverlayObjects: This is an optional module that overlays the identified objects (fibrosis) to the original image with an opacity of "0.5".

OverlayOutlines: If the "*OverlayObjects*" module is enabled, this module overlays the overlayed image with a color outline that by default is green. With these last optional modules, the precision of the pipeline can be seeing easily.

Threshold: A second threshold is used to add other strategies and methods to the ones in the first "*Threshold*" method. If not needed, this module can be disabled.

MeasureImageAreaOccupied: Measures the fibrotic area in the second threshold image. If the second "*Threshold*" module is disabled, change the image to measure from "ThresholdFibrosis" to "Threshold".

SaveImages: This module saves the images from the "*Threshold*" module ("ThresholdFibrosis" or "Threshold"). The prefix "-Morphometrics" can be changed, as well as the file format.

ExportToSpreadsheet: With this module the calculated fibrotic area can be saved into a spreadsheet. By default, the filename prefix "Morphometrics-" is used. To obtain the percentage of fibrosis, the columns needed for calculation are "AreaOccupied_AreaOccupied_ThresholdFibrosis" and "AreaOccupied_TotalArea_ThresholdFibrosis" and apply the following formula: %fibrosis = (AreaOccupied/TotalArea) * 100.

This pipeline is also available to be downloaded from:

https://cellprofiler.org/published-pipelines
